# Exploring the mechanisms underlying stroke volume variability reduction in a murine model of heart failure with reduced ejection fraction

**DOI:** 10.1371/journal.pone.0292687

**Published:** 2023-10-26

**Authors:** Gemma Fernández-Mendoza, Abraham Méndez-Fernández, Hugo J. Alves-Figueiredo, Gerardo García-Rivas, Moisés Santillán

**Affiliations:** 1 Departamento de Física, Escuela Superior de Física y Matemáticas, Instituto Politécnico Nacional, Ciudad de México, México; 2 The Institute for Obesity Research, Tecnologico de Monterrey, Monterrey, NL, Mexico; 3 Cátedra de Cardiología y Medicina Vascular, School of Medicine and Health Sciences, Tecnologico de Monterrey, Monterrey, NL, Mexico; 4 Unidad Monterrey, Centro de Investigación y de Estudios Avanzados, Apodaca NL, México; University of Rochester Medical Center, UNITED STATES

## Abstract

Heart failure with reduced ejection fraction (HFrEF) is accompanied by disregulation of cardiovascular function. Heart rate variability (HRV) is commonly used to assess autonomic dysfunction in HFrEF. However, analysis of stroke volume variability (SVV) may provide additional insights. We examined HRV and SVV in a mouse model of HFrEF. HFrEF mice exhibited reduced stroke volume and ejection fraction versus controls, confirming cardiac contractile dysfunction. HRV was preserved in HFrEF mice. However, SVV was markedly diminished, indicating dissociation between HRV and SVV regulation. Using a mathematical model, we propose that Frank-Starling mechanism abnormalities in HFrEF disrupt SVV independent of HRV. Assessing SVV could thus provide unique insights beyond HRV into cardiovascular control deficits in HFrEF.

## Introduction

Heart rate variability (HRV) has emerged as a noninvasive tool for assessing autonomic nervous system function [1, 2]. Decreased HRV has been associated with conditions like heart failure, diabetic neuropathy, and sudden infant death syndrome [3–6]. Stroke volume variability (SVV) has also gained recognition as an indicator of cardiovascular control and declining cardiac function [7, 8]. Liu et al. [9] demonstrated in healthy individuals that SVV time series have distinct power-spectrum characteristics from HRV, implying that SVV could provide additional insights into autonomic control beyond HRV alone. While the autonomic nervous system clearly regulates HRV, the mechanisms governing SVV are less established.

In healthy individuals, stroke volume fluctuates because inspiration increases venous return and left ventricular preload, augmenting stroke volume via the Frank-Starling mechanism. The opposite occurs during expiration, creating respiratory-induced variations in stroke volume [10]. However, in heart failure patients, this compensatory mechanism becomes impaired as reduced cardiac contractility limits the Frank-Starling response [11]. From this, we hypothesize that the SVV diminishes in heart failure and that this arises from alterations in the Frank-Starling mechanism.

To test this hypothesis, we will examine SVV in a murine model of heart failure with reduced ejection fraction. The goal is to shed light on the mechanisms underlying any SVV changes in heart failure. This could provide additional insights beyond HRV into how cardiac dysfunction affects cardiovascular control and advance our understanding of cardiovascular regulation in heart failure.

## Materials and methods

### Animal model of Heart Failure with Reduced Ejection Fraction (HFrEF)

Mice with induced heart failure were used to study heart rate and stroke volume variability. C57BL/6 male mice weighing 20–30 g were randomly divided into two groups: a control group (n = 14), and a heart-failure model group (n = 10). Over a span of 5 weeks, the mice in the model group were given ad libitum access to drinking water supplemented with 1% NaCl and 0.01% of N-nitroL-arginine methyl ester (L-NAME). Following one week of treatment commencement, a micro-osmotic pump was surgically implanted into the subdermal dorsal area to release angiotensin II (ANGII) at a rate of 0.7 mg/kg/day, based on a prior report [12]. On the other hand, the control group mice were provided plain water and underwent the same surgery but did not have a pump implanted.

All procedures were done with the assistance of veterinary staff and were previously reviewed and approved by the Institutional Animal Care and Use Committee of the School of Medicine of Tecnológico de Monterrey, in accordance with the Mexican regulation (NOM-062-ZOO-1999).

### Left-ventricle hemodynamics assessment

At 28 days post-implantation of the pump, left-ventricle hemodynamics was evaluated in vivo using pressure-volume (PV) analysis with an open-heart configuration and the ADV500 PV measurement system (Transonic Scisense, NY, USA). A 1.2 Fr PV catheter was utilized for this purpose, following the methodology described in a prior publication [13]. For each mouse examined, we assessed the following hemodynamic parameters: heart rate (HR), end systolic pressure (ESP), end diastolic pressure (EDP), peak pressure (*P*_*max*_), minimum pressure (*P*_*min*_), maximal rate of pressure rise ([*dP*/*dt*]_*max*_), minimum rate of pressure decrease ([*dP*/*dt*]_*min*_), maximum volume (*V*_*max*_), minimum volume (*V*_*min*_), end systolic volume (ESV), end diastolic volume (EDV), stroke volume (SV), cardiac output (CO), and ejection fraction (EF).

Anesthesia was first induced by 5% sevoflurane and then continued at a concentration of 3-5% during micro-osmotic pump implantation and P-V loop recording. Following invasive hemodynamics, animals were euthanized by cardiectomy under deep gas anesthesia. In order to minimize distress, animals received a humane endpoint euthanasia based on the professional assessment of the veterinary staff.

### Fibrosis and cellular hypertrophy assessment

After euthanizing the mice, their hearts were extracted and subjected to a semi-quantitative assessment of fibrosis and cellular hypertrophy, following [12]. The samples were stained with Masson’s Trichrome or hematoxylin and eosin, and microphotographs were captured using an Imager Z.1 Zeiss microscope equipped with an AxioCam HRm camera. The microphotographs were processed using the AxioVision software. The PV records of mice with hearts that showed no signs of fibrosis and cellular hypertrophy indicative of heart failure were excluded from the study.

### Apoptosis assessment

The activity of caspases 3/7 was assessed in heart homogenates via the Caspase Glo 3/7 (G8090, Promega) chemiluminescence assay. For each reaction, 100 μg of total protein was mixed with the reagent in a 1:1 volume proportion. Total volume was 100 μl. After one hour of incubation in room temperature conditions, chemiluminescence was recorded with the Synergy H1 microplate reader (BioTek). Luminescence was assumed to be proportional to caspases activity, which in its turn can be considered as a surrogate for apoptosis in heart cells.

### Variability analysis

A variety of techniques have been used to measure variability. The vast majority of them are founded on the concept of signal stationarity. However, the heart’s inherent non-stationary nature—which undergoes continuous physiological change to adapt to external stimuli—presents a significant challenge that could result in inaccurate results [14]. Although several signal preprocessing methodologies have been proposed to address these issues, nonlinear analysis-based strategies are commonly employed and appear to produce reliable results [4, 14–19]. One of them, used in different scientific domains is the Poincaré plot [18, 20, 21].

The Poincaré plot is constructed by plotting the value of a given variable at time *t* + 1 on the *x*-axis, against the value of the same variable at time *t* on the *y*-axis. Then, each point on the plot represents a pair of consecutive values of the variable, and the shape of the plot reflects the temporal dependencies and the non-linear dynamics of the system.

In a Poincaré plot, there are two measures of the scatter of the data points (termed SD1 and SD2), which are used to quantify the dynamics of the system. SD1 is the standard deviation of the perpendicular distance of the data points to the line of identity (i.e., the line *y* = −*x*). SD1 is also known as the short-term variability, and it reflects the magnitude of the fluctuations of the system at a short time scale. In the particular case of heart-beat duration time-series, SD1 is related to the parasympathetic nervous system activity, which regulates the beat-to-beat changes in heart rate. SD2, on the other hand, is the standard deviation of the distances of the data points to the center of gravity of the plot along the line *y* = *x*. SD2 is also known as the long-term variability, and it reflects the magnitude of the fluctuations of the system at a longer time scale. In the case of heart rate variability, SD2 is related to the sympathetic nervous system activity [22].

An algorithm was developed in Python to estimate the Poincaré-plot indices (SD1 and SD2) of heart-rate and stroke volume variability from time series data obtained experimentally. The algorithm has been made available for reference along with the experimentally recorded data at the following URL: https://github.com/moises-santillan/HeartVariability.

### Statistical analysis

To determine significant differences between groups, we employed statistical analyses such as Student’s t-tests and the Mann-Whitney U test, depending on the normality of the data. We utilized the ttest_ind or the mannwhitneyu function available in the scipy.stats library in Python for this purpose. We regarded results with p-values less than 0.05 as statistically significant.

## Results and discussion

### HFrEF murine model implementation

After establishing the murine model for HFrEF, we conducted an in vivo evaluation of left ventricular hemodynamics, following the procedures outlined in the Materials and Methods section. We assessed a range of parameters, including heart rate (HR), systolic and diastolic pressures, peak and minimum pressures, maximal and minimum rates of pressure change, systolic and diastolic volumes, stroke volume (SV), cardiac output (CO), and ejection fraction (EF). Notably, we observed a moderate positive correlation between stroke volume and ejection fraction in both the control and HFrEF groups, with correlation coefficients approximately equal to 0.65 and confidence levels exceeding 0.95. Detailed individual measurements are available in the online repository.

**Fig 1:**
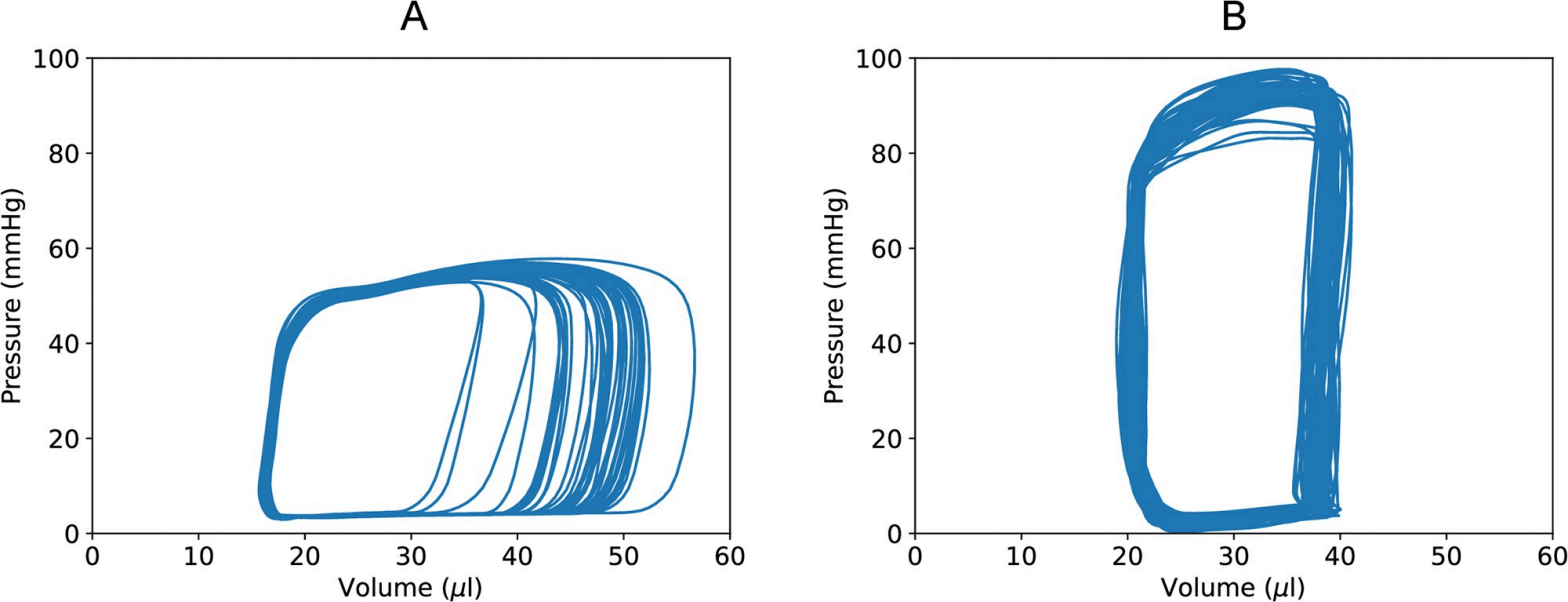
Representative left ventricular pressure-volume loops recorded from a control mouse (A) and a mouse with heart failure induced by NaCl, L-NAME and angiotensin II (B). The heart failure mouse exhibits characteristic abnormalities in the pressure-volume relationship, including increased end-systolic pressure, reduced stroke volume, and diminished ejection fraction. These hemodynamic alterations are consistent with heart failure with reduced ejection fraction.

The model group exhibited canonical hemodynamic changes associated with HFrEF, including augmented end-systolic pressure, decreased stroke volume (SV) and cardiac output (CO), and reduced ejection fraction (EF) compared to controls (Table 1). Representative pressure-volume loops in Fig 1 further illustrate the alterations in ventricular function between control and HFrEF mice.

**Table 1:** Average hemodynamic parameters and standard deviations for control and HFrEF mice. Statistically significant differences between control (n = 14) and HFrEF (n = 10) mice are denoted by asterisks (*p* < 0.05). Compared to controls, HFrEF mice displayed characteristic abnormalities such as increased end-systolic pressure, reduced stroke volume and cardiac output, and decreased ejection fraction.

	Control (Avg ± Std)	HFrEF (Avg ± Std)
HR (bpm)	481.14 ± 44.74	483.60 ± 37.22
ESP (mmHg) *	54.05 ± 12.22	82.95 ± 18.75
EDP (mmHg)	4.53 ± 2.25	5.94 ± 2.77
*P*_*max*_ (mmHg) *	60.93 ± 11.78	92.60 ± 19.18
*P*_*min*_ (mmHg)	2.58 ± 2.10	3.12 ± 1.73
[*dP*/*dt*]_*max*_ (mmHg/s) *	4309.12 ± 973.09	5702.65 ± 1391.61
[*dP*/*dt*]_*min*_ (mmHg/s)	-3829.84 ± 1270.54	-5546.37 ± 1610.70
*V*_*max*_ (μl) *	41.28 ± 9.31	27.82 ± 11.27
*V*_*min*_ (μl)	12.48 ± 5.75	11.58 ± 4.98
ESV (μl)	15.33 ± 6.45	13.36 ± 5.46
EDV (μl) *	39.13 ± 9.04	25.45 ± 9.46
SV (μl) *	23.80 ± 5.48	12.09 ± 4.80
CO (μl/min) *	11471.31 ± 2879.14	5894.72 ± 2373.94
EF (%) *	61.74 ± 11.76	46.97 ± 10.05

**Fig 2:**
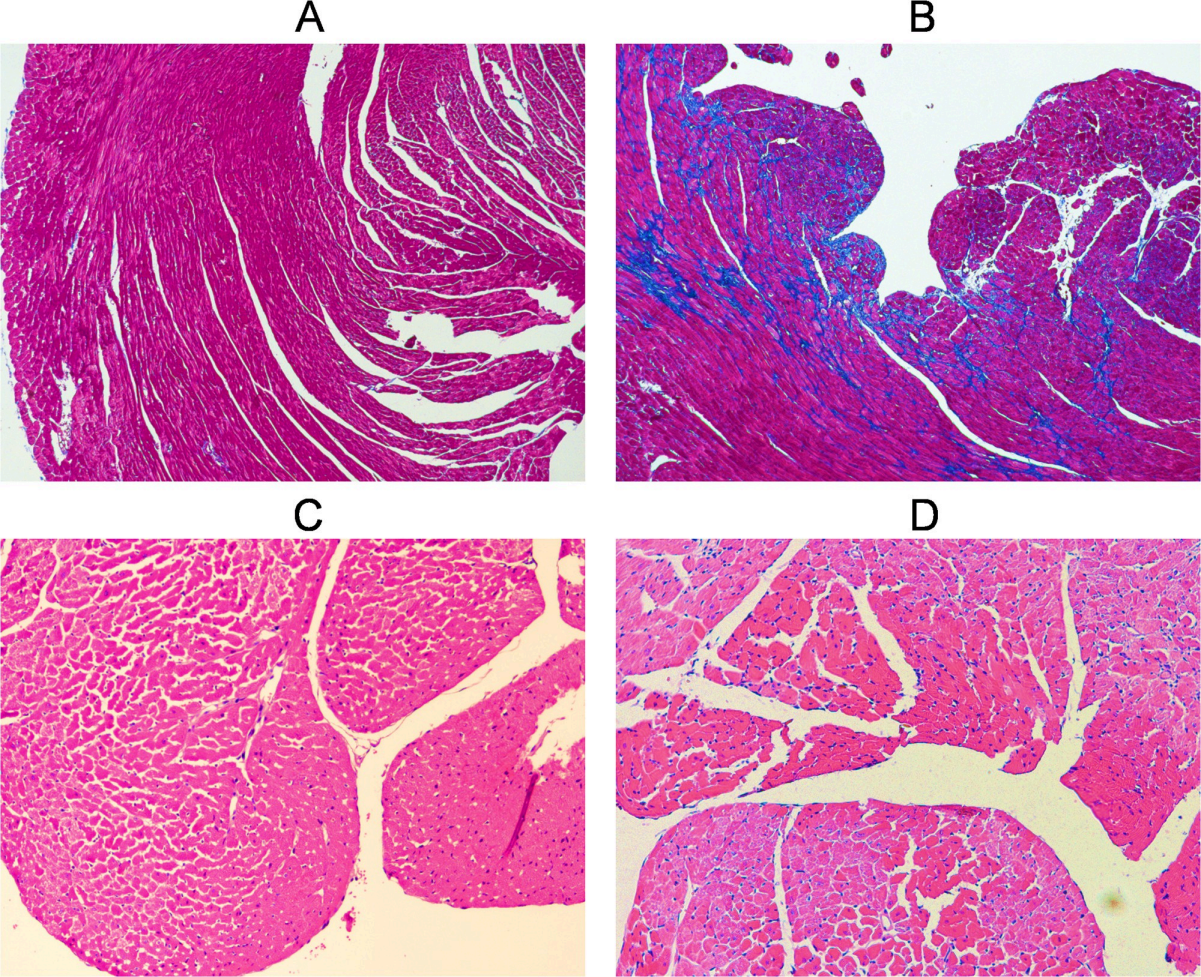
Representative photomicrographs of myocardial fibrosis (A,B) and cellular hypertrophy (C,D) in control (A,C) and HFrEF (B,D) mice. Masson’s trichrome staining shows increased interstitial fibrosis (blue) in the HFrEF heart (B) compared to control (A). Hematoxylin and eosin staining reveals enlarged cardiomyocytes with increased cytoplasmic area in the HFrEF heart (D) compared to control (C). Each image corresponds to a square area of 500 μm X 500 μm.

After hemodynamic assessment, the hearts were examined histologically. Fig 2 shows representative images of enhanced fibrosis and cellular hypertrophy in HFrEF hearts compared to controls.

**Fig 3:**
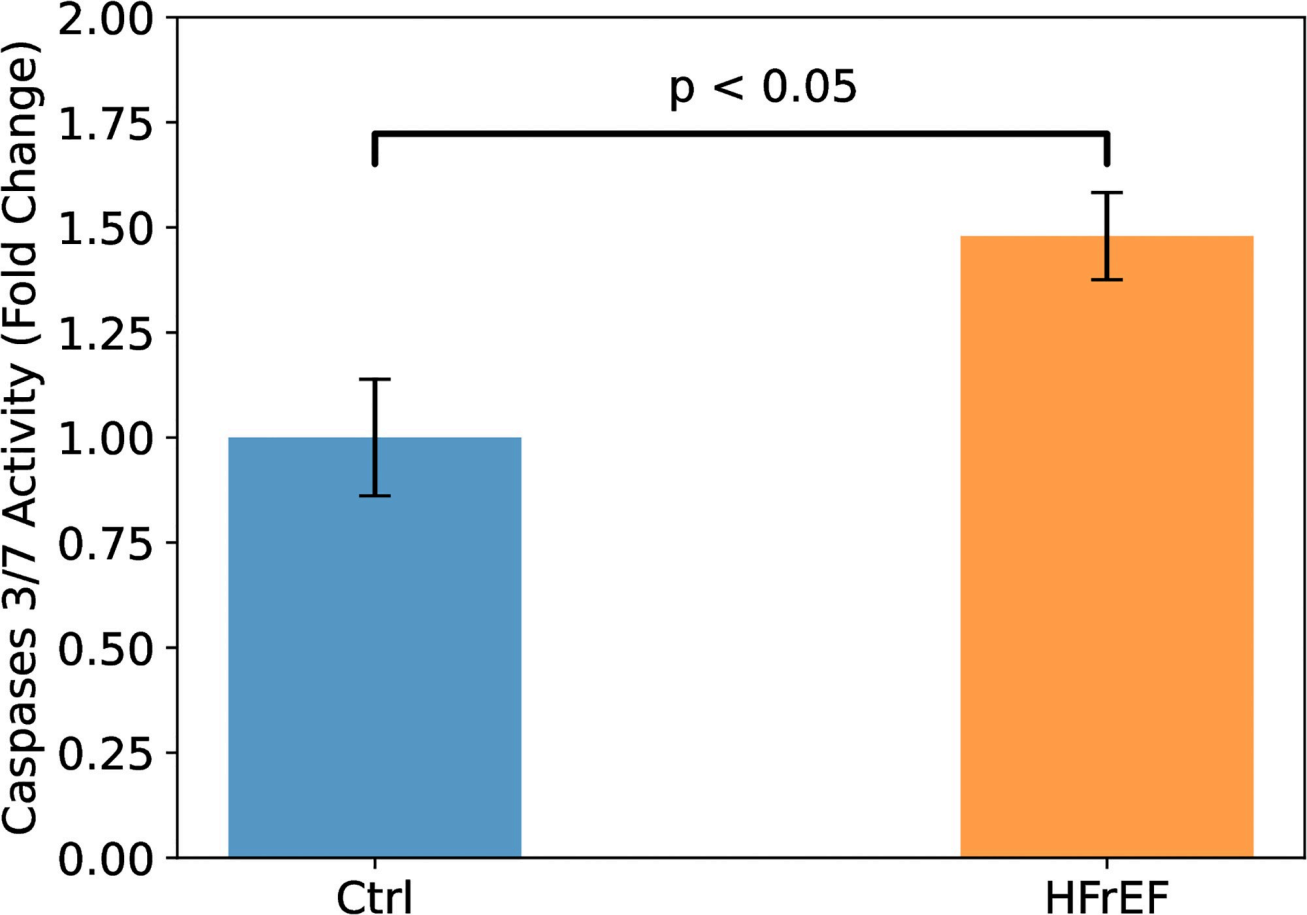
Plot of normalized Caspases 3/7 activity in heart homogenates of control (n = 5) and HFrEF (n = 9) mice. HFrEF mice exhibited significantly increases Caspases 3/7 activity, indicating intensified apoptosis. Brackets denote statistically significant differences at *p* < 0.05. Error bars indicate standard error of the mean.

Apoptosis was quantified in homogenates from both control (n = 5) and HFrEF (n = 9) mice. As depicted in Fig 3, the findings reveal a noteworthy 50% surge in Caspases 3/7 activity within HFrEF hearts when compared to the control group, providing clear evidence of intensified apoptosis.

In summary, the implemented protocol successfully generated a murine model exhibiting cardiovascular abnormalities resembling human HFrEF. This provides a suitable platform for studying heart rate and stroke volume variability in the context of heart failure and reduced ejection fraction.

### Heart rate and stroke volume variability analysis

**Fig 4:**
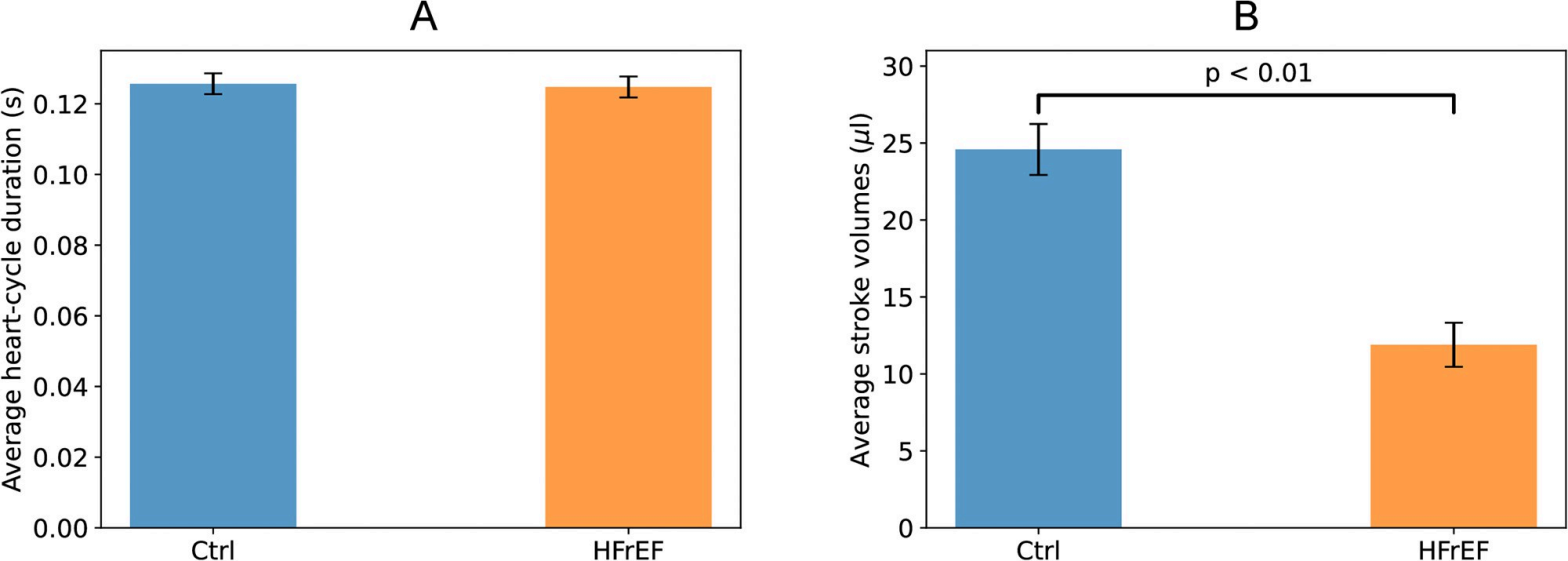
Average cardiac cycle duration (A) and stroke volume (B) for control (n = 14) and HFrEF (n = 10) mice groups. HFrEF mice exhibited significantly reduced stroke volumes compared to controls, indicating impaired cardiac contractility. However, average cycle durations were not significantly different between groups under anesthesia. Brackets denote statistically significant differences at *p* < 0.05. Error bars indicate standard error of the mean.

We first examined heart rate by calculating the average cycle duration from the time series of consecutive cardiac cycles for each mouse. As shown in Fig 4A, there was no significant difference in heart rate between the control and HFrEF groups, contradicting previous clinical findings that heart failure patients tend to have higher resting heart rates [3]. However, the employed anesthesia is known to affect heart rate [23, 24], which may explain this discrepancy. Stroke volume was significantly reduced in the HFrEF group compared to controls (Fig 4B), consistent with impaired cardiac contractility.

**Fig 5:**
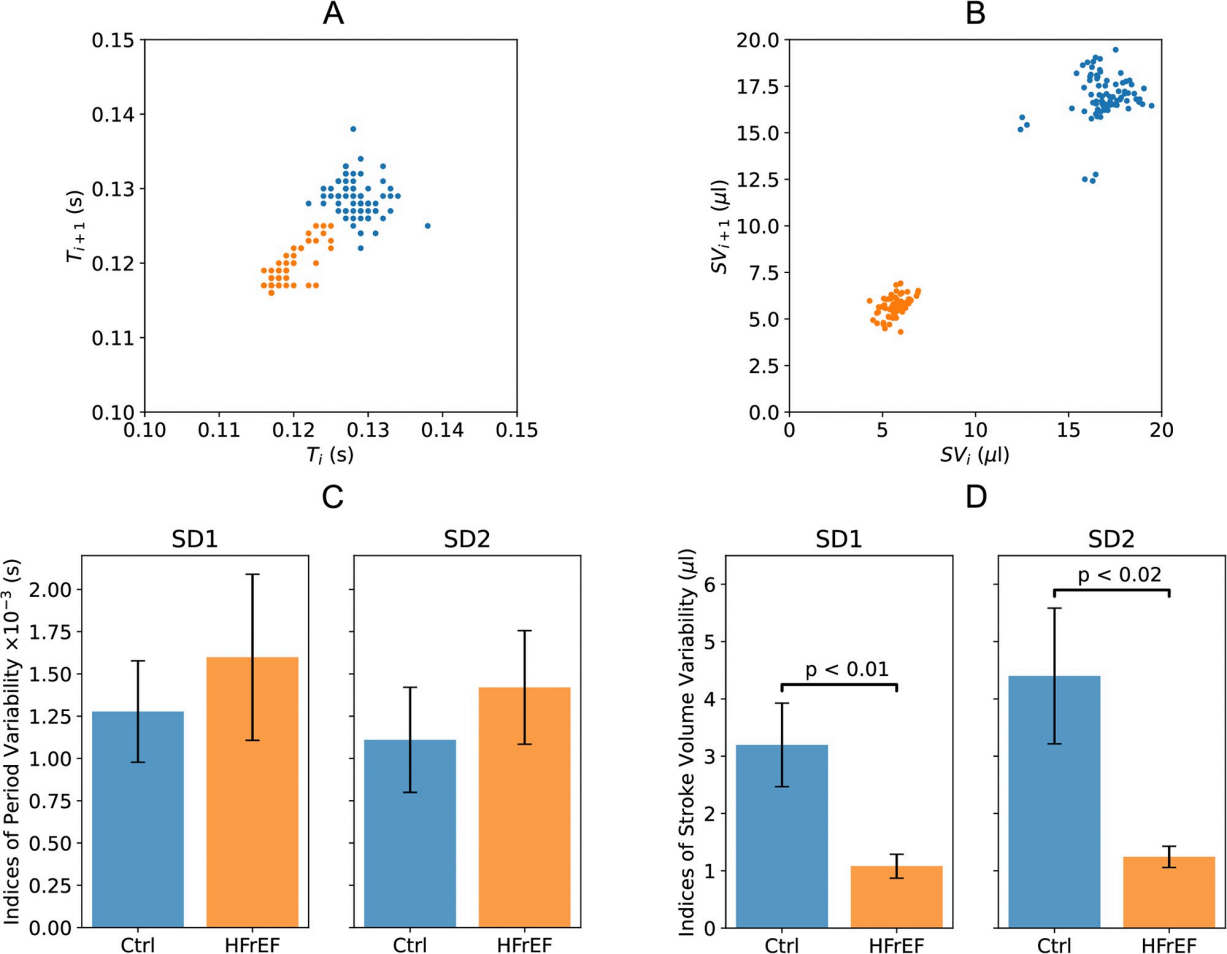
Representative Poincaré plots of heart rate (A) and stroke volume (B) for control (blue) and HFrEF (orange) mice. Quantified Poincaré plot standard deviations SD1 and SD2 for heart rate (C) and stroke volume (D) in control (n = 14) and HFrEF (n = 10) mice. Heart rate variability (SD1 and SD2) was preserved in HFrEF mice compared to controls. In contrast, stroke volume variability (SD1 and SD2) was significantly reduced in HFrEF mice relative to controls. This indicates dissociation between heart rate and stroke volume regulation in the HFrEF model. Statistically significant differences between groups denoted by brackets at *p* < 0.05. Error bars show standard error of the mean.

To assess variability, we constructed Poincaré plots and quantified short-term (SD1) and long-term (SD2) variability for cycle duration and stroke volume. Representative Poincaré plots and quantified variability indices are displayed in Fig 5. Notably, while cycle duration variability was unchanged in HFrEF mice, both SD1 and SD2 were significantly reduced for stroke volume. This indicates that stroke volume variability is diminished in heart failure through mechanisms distinct from heart rate control.

The preservation of heart rate variability suggests equal autonomic regulation both groups of our anesthetized model. However, the reduction in stroke volume fluctuations suggests that contractile dysfunction in HFrEF may disrupt cardiovascular variability through effects on other mechanisms like the Frank-Starling mechanism. We explore this further through mathematical modeling. Overall, our findings reveal dissociation between heart rate and stroke volume variability in heart failure, warranting additional investigation into the distinct mechanisms governing these two aspects of cardiovascular variability.

### Investigating SVV through a mathematical model

**Fig 6:**
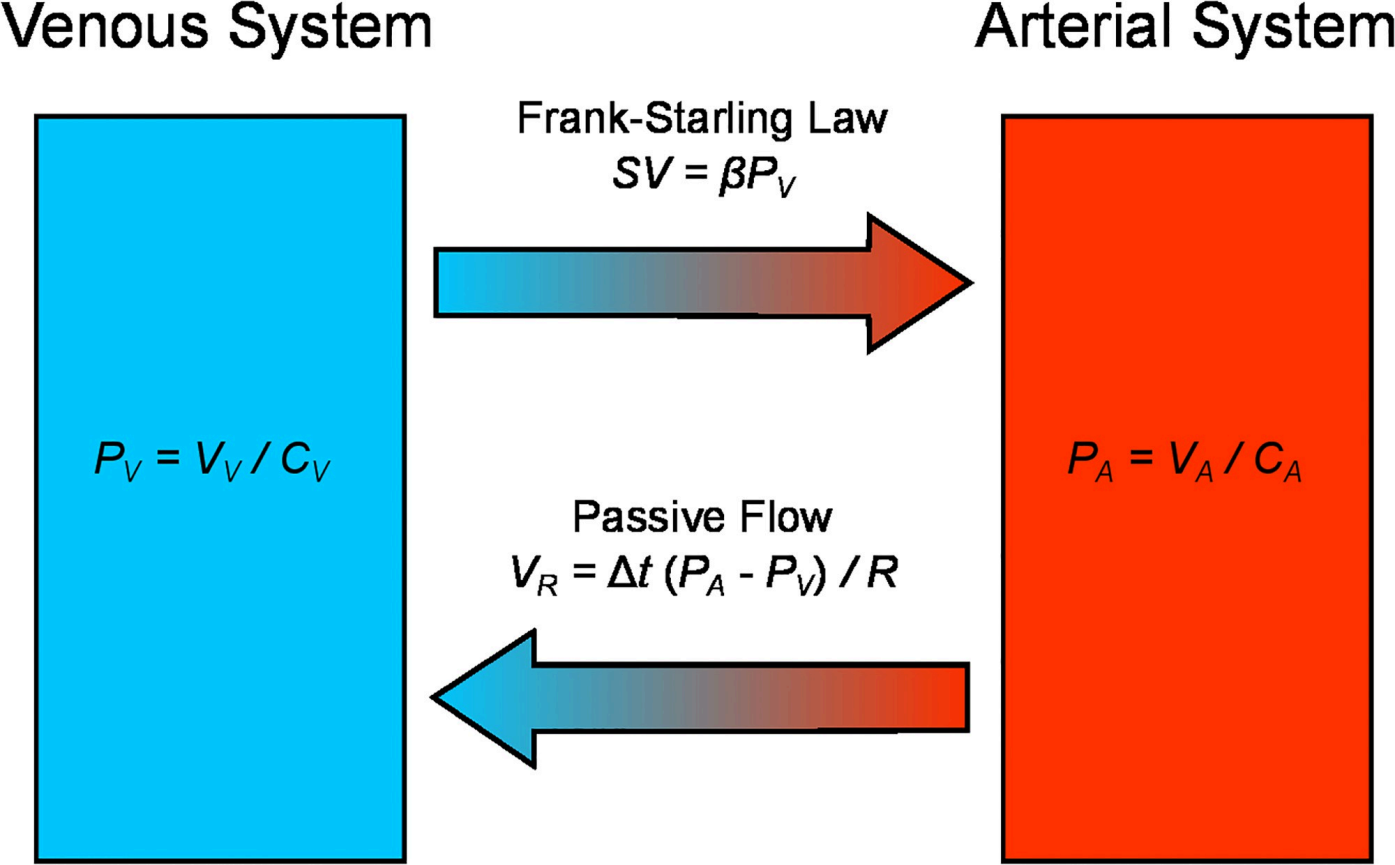
Schematic of the mathematical model representing the Frank-Starling mechanism of cardiovascular regulation. The model consists of arterial (*A*) and venous (*V*) compartments connected via passive flow (*R*) and a contractile element representing the heart that pumps blood based on venous pressure (*β*). We propose that abnormalities in this mechanism in HFrEF underlie the reduction in stroke volume variability independently of heart rate control.

We use a model of the cardiovascular system introduced by Upton et al. [25], which is schematically represented in Fig 6. It separates the circulating blood into arterial and venous compartments. The pressure (*P*_*x*_) and blood volume (*V*_*x*_) in each compartment are related by the corresponding compliance (*C*_*x*_):

$$\begin{array}{c} P_{x}=\frac{V_{x}}{C_{x}}, \end{array}$$

where subindex *x* can take the values *A* or *V* to represent the arterial and venous compartments. The flow of blood from the arterial compartment to the venous compartment is assumed to be passive. Hence, the blood volume exchanged during period Δ*t* is:

$$\begin{array}{c} V_{R}=\Delta t(P_{A}-P_{V})/R, \end{array}$$

where *R* denotes the peripheral vascular resistance. Finally, to account for the Frank-Starling Law [26], the volume of blood transferred from the venous compartment to the arterial compartment by the heart pumping (stroke volume), is assumed to depend linearly on the venous pressure:

$$\begin{array}{c} SV=\beta P_{V}. \end{array}$$

This is the model’s most daring assumption, as it integrates the pulmonary circulation of blood and its passage through the heart auricles and ventricles in a single step.

We now employ the Upton et al. model to investigate how the system’s current state influences its evolution over successive cycles. Let *V*_*V*,*i*_ and *P*_*V*,*i*_ denote the venous volume and pressure at the end of the *i*-th heart cycle. The stroke volume in the next cycle is then:

$$\begin{array}{c} SV_{i+1}=\beta P_{V,i}. \end{array}$$

With this, the venous and arterial volumes after the heart stroke are:

$$\begin{array}{cc} V_{V,i+1/2} & =V_{V,i}-SV_{i+1}\\ V_{A,i+1/2} & =V_{A,i}+SV_{i+1}. \end{array}$$

After some algebra, these can be rewritten as:

$$\begin{array}{cc} V_{V,i+1/2} & =V_{V,i}(1-\frac{\beta }{C_{V}})\\ V_{A,i+1/2} & =V_{T}-V_{V,i}(1-\frac{\beta }{C_{V}}), \end{array}$$

where *V*_*T*_ is the total blood volume, assumed constant since the system is closed. After the heart stroke, a volume *V*_*R*,*i*+1_ of blood passively diffuses back to the venous compartment:

$$\begin{array}{c} V_{R,i+1}=\frac{1}{R}[\frac{V_{T}}{C_{A}}-V_{V,i}(1-\frac{\beta }{C_{V}})(\frac{1}{C_{A}}+\frac{1}{C_{V}})]. \end{array}$$

The venous and arterial volumes at the end of the *i* + 1-th cycle are:

$$\begin{array}{cc} V_{V,i+1} & =V_{V,i}+V_{R,i+1}\\ V_{A,i+1} & =V_{A,i}-V_{R,i+1}. \end{array}$$

This results in:

$$\begin{array}{c} V_{V,i+1}=V_{V,i}(1-\frac{\beta }{C_{V}})[1-\frac{1}{R}(\frac{1}{C_{A}}+\frac{1}{C_{V}})]+\frac{V_{T}}{RC_{A}}. \end{array}$$

Taking *SV*_*i*+1_ = *βV*_*V*,*i*_/*C*_*V*_, it follows that:

$$\begin{array}{c} SV_{i+1}=A\;SV_{i}+B, \end{array}$$

where

$$\begin{array}{cc} A & =(1-\frac{\beta }{C_{V}})[1-\frac{1}{R}(\frac{1}{C_{A}}+\frac{1}{C_{V}})]\\ B & =\frac{V_{T}\beta }{RC_{A}C_{V}}. \end{array}$$

This recursive equation for stroke volume converges to:

$$\begin{array}{c} \bar{SV}=\frac{B}{1-A}, \end{array}$$

when |A|<1.

When noise is present, the recursive equation for stroke volume predicts that stroke volume variability will be lower for smaller values of A. Since A depends inversely on *β*, the Frank-Starling parameter, the reduced stroke volume variability could arise from changes in the value of *β*. Indeed, despite earlier controversy, Holubarsch [11] demonstrated that the Frank-Starling mechanism persists in failing human hearts, though with reduced cardiac contractility. Factors like fibrosis, hypertrophy and apoptosis contribute to this decline in contractility, thereby diminishing the Frank-Starling response. This reduction in contractility, mediated in part by ventricular remodeling, may explain the change of parameter *β* in our model. Thus, our model proposes that changes in *β* induced by heart failure help explain the decreased stroke volume variability observed in our mouse model of HFrEF. Further research is still needed to fully elucidate how fibrosis, hypertrophy, apoptosis and other factors integrate with the proposed Frank-Starling abnormalities.

## Conclusions

In this study, we investigated heart rate and stroke volume variability in a mouse model of heart failure with reduced ejection fraction. Under anesthesia, heart rate and heart rate variability were unchanged in HFrEF mice compared to controls. However, stroke volume and stroke volume variability were significantly reduced.

These findings contradict previous clinical studies showing diminished heart rate variability in heart failure patients. We speculate that anesthesia may attenuate autonomic dysfunction in our model, preserving heart rate variability. However, reduced stroke volume fluctuations imply that alternate mechanisms are disrupting cardiovascular control in HFrEF.

Through mathematical modeling, we proposed that abnormalities in the Frank-Starling mechanism could explain the selective reduction in stroke volume variability. Changes in ventricular compliance and contractility in heart failure may impair the Frank-Starling response that normally mediates respiratory-induced stroke volume fluctuations.

Our study has limitations, including the effects of anesthesia and invasive hemodynamic measurement. Further research using less invasive approaches in conscious animal models is needed to validate the relationship between stroke volume variability and cardiac contractile function. Nonetheless, our findings suggest that assessing SVV could provide unique insights beyond HRV into cardiovascular control deficits in HFrEF.

Further research is warranted to validate the relationship between SVV and cardiac contractility. While our findings in a mouse model shed light on potential mechanisms governing cardiovascular variability, future studies should extend this work to human patients. Characterizing SVV in clinical populations would help confirm the translational relevance of our results. Additionally, analyzing other heart failure induction models beyond just angiotensin II infusion could help elucidate the generalizability and nuances of the observed SVV changes.
